# 1-Nitropyrene Induced Reactive Oxygen Species–Mediated Apoptosis in Macrophages through AIF Nuclear Translocation and AMPK/Nrf-2/HO-1 Pathway Activation

**DOI:** 10.1155/2021/9314342

**Published:** 2021-07-13

**Authors:** Chun-Hung Su, Yung-Chuan Ho, Min-Wei Lee, Ching-Chi Tseng, Shiuan-Shinn Lee, Ming Kun Hsieh, Hsin-Hung Chen, Chien-Ying Lee, Sheng-Wen Wu, Yu-Hsiang Kuan

**Affiliations:** ^1^Department of Internal Medicine, Chung Shan Medical University Hospital, Taichung, Taiwan; ^2^Department of Internal Medicine, School of Medicine, Chung Shan Medical University, Taichung, Taiwan; ^3^Institute of Medicine, Chung Shan Medical University, Taichung, Taiwan; ^4^School of Medical Applied Chemistry, Chung Shan Medical University, Taichung, Taiwan; ^5^A Graduate Institute of Microbiology and Public Health, National Chung Hsing University, Taichung, Taiwan; ^6^Aerospace Center Hospital, Peking University, Beijing, China; ^7^School of Public Health, Chung Shan Medical University, Taichung, Taiwan; ^8^Division of Endocrinology and Metabolism, Department of Internal Medicine, Asia University Hospital, Taichung, Taiwan; ^9^School of Medicine, Institute of Medicine and Public Health, Chung Shan Medical University, Taichung, Taiwan; ^10^Chung Sheng Clinic, Nantou, Taiwan; ^11^Department of Pharmacology, School of Medicine, Chung Shan Medical University, Taichung, Taiwan; ^12^Department of Pharmacy, Chung Shan Medical University Hospital, Taichung, Taiwan; ^13^Division of Nephrology, Department of Internal Medicine, Chung Shan Medical University Hospital, Taichung, Taiwan; ^14^The School of Medicine, Chung Shan Medical University, Taichung, Taiwan

## Abstract

1-Nitropyrene (1-NP), one of the most abundant nitropolycyclic aromatic hydrocarbons (nitro-PAHs), is generated from the incomplete combustion of carbonaceous organic compounds. 1-NP is a specific marker of diesel exhaust and is an environmental pollutant and a probable carcinogen. Macrophages participate in immune defense against the invasive pathogens in heart, lung, and kidney infection diseases. However, no evidence has indicated that 1-NP induces apoptosis in macrophages. In the present study, 1-NP was found to induce concentration-dependent changes in various cellular functions of RAW264.7 macrophages including cell viability reduction; apoptosis generation; mitochondrial dysfunction; apoptosis-inducing factor (AIF) nuclear translocation; intracellular ROS generation; activation of the AMPK/Nrf-2/HO-1 pathway; changes in the expression of BCL-2 family proteins; and depletion of antioxidative enzymes (AOE), such as glutathione peroxidase (GPx), catalase (CAT), and superoxide dismutase (SOD) These results indicate that 1-NP induced apoptosis in macrophages through AIF nuclear translocation and ROS generation due to mitochondrial dysfunction and to the depletion of AOE from the activation of the AMPK/Nrf-2/HO-1 pathway.

## 1. Introduction

1-Nitropyrene (1-NP) is a nitropolycyclic aromatic hydrocarbon (nitro-PAH), a class of environmental pollutants generated from the incomplete combustion of carbonaceous organic fuels, biomass, and other compounds [1]. 1-NP is a highly specific marker of diesel exhaust. Various studies have detected 1-NP in the environment and in foods, including in soil, road dust, rice, cabbage, and the atmosphere [2, 3]. The high lipid solubility of 1-NP allows it to permeate the gastrointestinal system, respiratory system, and skin [4]. 1-NP is one of the most abundant nitro-PAHs in urban ambient air and is a major contributor to mutagenic and carcinogenic effects [5–7]. The International Agency for Research on Cancer (IARC) has classified 1-NP as a group 2A carcinogen, indicating that it is probably carcinogenic to humans [8]. The 1-NP exposures experienced by ambient the concentrations in air ranged from 10 to 1000 pg/m^3^ in urban areas. The concentration of 1-NP in the rural and remote areas with low traffic intensity ranges from 1 to 100 pg/m^3^ in the whole world. Concentrations of 1-NP tend to be higher in winter than in summer [8]. Up to now, there is no evidence showing the human carcinogen induced by 1-NP [8]. However, several diseases caused by the long-term exposure lead to accumulation of 1-NP in the animal model. The liver, lung, and mammary gland carcinomas are induced by 1-NP at the concentration range from 25 to 100 *μ*M/kg for long-term exposure above 12 weeks in animals [8]. Apoptosis plays a significant role in pathogenesis, metagenesis, and tumorigenesis through mitochondrial dysfunction [9]. 1-NP induces apoptosis in liver epithelial Hepa1c1c7 cells, hepatoma HepG2 cells, bronchial epithelial BEAS-2B cells, and type II pulmonary epithelial A549 cells [6, 10–12].

Macrophages, which differentiate from monocytes, are a group of mononuclear phagocytes that participate in immune defense against invasive pathogens in heart, lung, and kidney infections [13]. Alveolar macrophages are the predominant resident phagocytes in the alveolar air space. When activated, they defend against inhaled pathogens, such as environmental pollutants and invasive bacteria, and lung trauma [13, 14]. The excess activation of macrophages can result in inflammatory responses and lead to cytotoxicity and apoptosis [15, 16]. Mitochondrial disruption plays a key role in macrophage apoptosis [14, 17]. Several molecular mechanisms participate in mitochondrial disruption which include the expression of the Bcl-2 family; translocations of apoptosis-inducing factor (AIF) and cytochrome c; and depletion of antioxidative enzymes (AOEs), such as glutathione peroxidase (GPx), catalase (CAT), superoxide dismutase (SOD), and heme oxygenase-1 (HO-1) [14, 18–20]. Recently, we have reported that cytotoxicity and genotoxicity were induced by 1-NP by poly (ADP-ribose) polymerase-1 (PARP-1) cleavage via caspase-3 and -9 activation through cytochrome c release from mitochondria and its upstream p53-dependent pathway in macrophages [21]. However, no evidence has indicated 1-NP-induced apoptosis in macrophages. Therefore, the current study examined cell viability and apoptosis in macrophages exposed to 1-NP and analyzed the mechanism of action.

## 2. Materials and Methods

### 2.1. Materials

Dulbecco's modified Eagle's medium (DMEM), trypsin, fetal bovine serum (FBS), penicillin, streptomycin, and fungizone were obtained from Hyclone (Logan, UT, USA). 1-Nitropyrene (1-NP), 2′,7′-dichlorodihydrofluorescein diacetate (DCFH-DA), 3-(4,5-dimethylthiazol-2-yl)-2,5-diphenyl-tetrazolium bromide (MTT), phosphate-buffered saline (PBS), dimethyl sulfoxide (DMSO), and other reagents of analytical grade were obtained from Sigma-Aldrich (St. Louis, MO, USA). Tetraethylbenzimidazolylcarbocyanine iodide (JC-1), catalase (CAT) assay kit, superoxide dismutase (SOD) assay kit, and glutathione peroxidase (GPx) assay kit were obtained from Cayman (Ann Arbor, MI, USA). Mitochondrial permeability transition pore (MPTP) assay kit was obtained from BioVision (Milpitas, CA, USA). Primary antibodies for the detection of AIF, cytochrome c, Bcl-2, Bcl-xL, Bad, Bax, Bid, HO-1, Nrf2, P-AMPK, AMPK, COX-IV, GAPDH, and *β*-actin were obtained from Santa Cruz (Santa Cruz, CA, USA). Secondary antibodies for horseradish peroxidase- (HRP-) conjugated mouse anti-rabbit IgG or goat anti-mouse IgG were purchased from Jackson ImmunoResearch Laboratories (Baltimore, MD, USA). Dissolve 1-NP in DMSO, in which the final concentration was not higher than 0.05% (*v*/*v*).

### 2.2. Cell Culture

The murine macrophage-like cell line RAW264.7 (BCRC 6001) was obtained from the Bioresource Collection and Research Centre (Taiwan). All cells were grown as monolayer cultures at 37°C in 5% CO_2_ using DMEM supplemented with 1% penicillin, streptomycin, fungizone, and 10% FBS. Cell passaging was conducted using 0.05% trypsin with 0.53 mM EDTA [20]. After seeding, the cells were incubated with 1-NP at the concentrations of 0, 3, 10, 30, and 50 *μ*M for 6, 12, 24, and 48 h for cell viability assay. On the other hand, the cells were incubated with 1-NP at the concentrations of 0, 3, 10, 30, and 50 *μ*M for 24 h used for other experimental assays.

### 2.3. Cell Viability Assay

3-(4,5-Dimethylthiazol-2-yl)-2,5-diphenyltetrazolium bromide (MTT) assay was used to evaluate cell viability [22]. The RAW264.7 cells were seeded at a density of 5 × 10^4^ cells/well in 96-well plates for 12 h. After replacing the serum- and phenol red-free medium with culture medium, the cells were exposed to 0, 3, 10, 30, and 50 *μ*M concentrations of 1-NP for 6, 12, 24, and 48 h. After treatment, 5 mg/mL MTT was added into each well. After 4 h at 37°C, the supernatant was carefully removed. DMSO was added into each well. Optical density (OD) was measured on a microplate reader (Synergy HT Multi-Mode Microplate Reader, BioTek, Winooski, VT) at a test wavelength of 550 nm.

### 2.4. Flow Cytometric Analysis of Necrosis and Apoptosis

Differentiation of apoptosis and necrosis was performed on a BD Accuri C6 flow cytometer (San Jose, CA, USA) using an FITC-Annexin V/PI apoptosis detection kit. The RAW264.7 cells were seeded at a density of 5 × 10^5^ cells/well in 24-well plates for 12 h. After replacing the serum- and phenol red-free medium with culture medium, the cells were exposed to 0, 3, 10, 30, and 50 *μ*M concentrations of 1-NP for 24 h. After 10^5^ cell collection, the apoptosis and necrosis were identified through dual staining with FITC-Annexin V and PI staining solution in the dark at room temperature for 15 min, as described previously [22]. Early apoptotic cells were Annexin V-positive and PI-negative (FITC-Annexin V+/PI−), late apoptotic cells were Annexin V- and PI-double-positive (FITC-Annexin V+/PI+), necrotic cells were Annexin V-negative and PI-positive (FITC-Annexin V−/PI+), and surviving cells were Annexin V- and PI-double-negative (FITC-Annexin V−/PI−).

### 2.5. Mitochondrial Membrane Potential (MMP) Assay

Mitochondrial membrane potential (MMP) was assessed using mitochondrial membrane potential assay dye JC-1, according to the manufacturer's protocol, as described previously [22]. After the 5 × 10^5^ cells were treated with 1-NP at various concentrations for 24 h, they were washed twice with PBS and incubated with JC-1 dye in serum-free medium for 30 min at 37°C. After washing, the cells were analyzed using the BD Accuri C6 flow cytometer.

### 2.6. Mitochondrial Permeability Transition Pore (MPTP) Assay

Mitochondrial permeability transition pore (MPTP) was assessed using the commercial assay kit according to the manufacturer's protocol. After the 5 × 10^5^ cells were treated with 1-NP at various concentrations for 24 h, they were washed and incubated with MPTP staining dye in serum-free medium for 15 min at 37°C. And then, the cells were incubated with 1 mM CoCl_2_ for 15 min at 37°C. After washing, the cells were analyzed using the BD Accuri C6 flow cytometer.

### 2.7. Measurement of Intracellular ROS Concentration

Intracellular ROS generation was evaluated using DCFH-DA, per the method of our previous study [14]. After the 5 × 10^5^ cells were treated with 1-NP at various concentrations for 24 h, the cells were incubated with DCFH-DA for 30 min at 37°C. After washing with PBS, the fluorescence was measured in a microplate reader at an excitation wavelength of 488 nm and emission wavelength of 515 nm.

### 2.8. Cell Fractionation and Western Blot Assay

The levels of protein expression from whole cells and subcellular fractions were measured using western blot assay, per a previously described method [14]. The RAW264.7 cells were seeded at a density of 5 × 10^6^ cells/well in a 10 cm dish for 12 h. After replacing the serum- and phenol red-free medium with culture medium, the cells were exposed to 0, 3, 10, 30, and 50 *μ*M concentrations of 1-NP for 24 h. After cell collection, protein from whole cells was extracted in lysis buffer (25 mM Tris-HCl at pH 7.6, 1 mM phenylmethylsulphonyl fluoride, 150 mM sodium chloride, 1% Nonidet P-40, 1 mM sodium orthovanadate, 10% glycerol, 0.1% SDS, and phosphatase and protease inhibitors). The fraction protein, containing cytosol, mitochondria, and nuclei, was isolated from cells using a cytoplasmic and nuclear protein extraction kit and mitochondria extraction kit. The protein content of the supernatant was determined using Bradford assay. Equal amounts of proteins were incubated with 5X sample buffer, separated by 7.5%–12.5% SDS-PAGE, and electrophoretically transferred onto polyvinylidene difluoride membranes. The membranes were blocked with 5% skimmed milk for 1 h at room temperature. They were then incubated with the indicated primary antibodies (AIF, Bcl-2, Bcl-xL, Bad, Bax, Bid, HO-1, Nrf2, P-AMPK, AMPK, and *β*-actin) with 0.5% skim milk overnight at 4°C and then with the secondary antibody for 1 h at room temperature. Finally, the membranes were visualized with protein densitometry analysis using the electrochemiluminescence (ECL) detection system.

### 2.9. Measurement of AOE Activities

The RAW264.7 cells were seeded at a density of 10^6^ cells/well in 6-well plates for 12 h. After replacing the serum- and phenol red-free medium with culture medium, the cells were exposed to 0, 3, 10, 30, and 50 *μ*M concentrations of 1-NP for 24 h. After cell collection, the AOE activities which include GPx, CAT, and SOD were assayed with the respective detection kits according to the manufacturer's instructions [14].

### 2.10. Statistical Analysis

Data of the results were representative of three independent experiments in western blot assay, fourth independent experiments in measurement of AOE activities, fifth independent experiments in cell viability assay, necrosis and apoptosis analysis, MMP assay, and measurement of intracellular ROS concentration. The values of the results were representative in terms of the mean ± standard deviation (SD). All data were analyzed in SPSS software. Multiple group comparisons were performed using one-way ANOVA followed by Bonferroni's post hoc test. *P* < 0.05 indicated statistical significance for all tests.

## 3. Results

### 3.1. Effects of 1-NP on Cell Viability in RAW264.7 Macrophages

**Figure 1:**
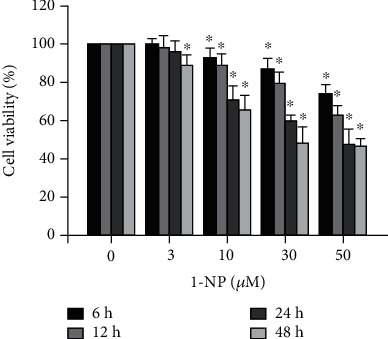
1-Nitropoyrene (1-NP) reduced cell viability in RAW264.7 macrophages. The cells were incubated with 1-NP at concentrations of 0, 3, 10, 30, and 50 *μ*M for 6, 12, 24, and 48 h at 37°C. Cell viability was measured using the MTT assay. Data are expressed as mean ± SD (*n* = 5). ^∗^*P* < 0.05 was considered significant compared with the control group.

The cell viabilities of RAW264.7 macrophages incubated with 0, 3, 10, 30, or 50 *μ*M concentrations of 1-NP for 6, 12, 24, and 48 h were monitored using MTT colorimetric assay (Figure 1). 1-NP reduced cell viability in a concentration- and time-dependent manner. The survival rate was significantly lower in RAW264.7 cells incubated with 3 *μ*M of 1-NP for 48 h and in cells incubated with 10 *μ*M of 1-NP for 12 h (*P* < 0.05).

### 3.2. Effects of 1-NP on Apoptosis and Necrosis in RAW264.7 Macrophages

**Figure 2:**
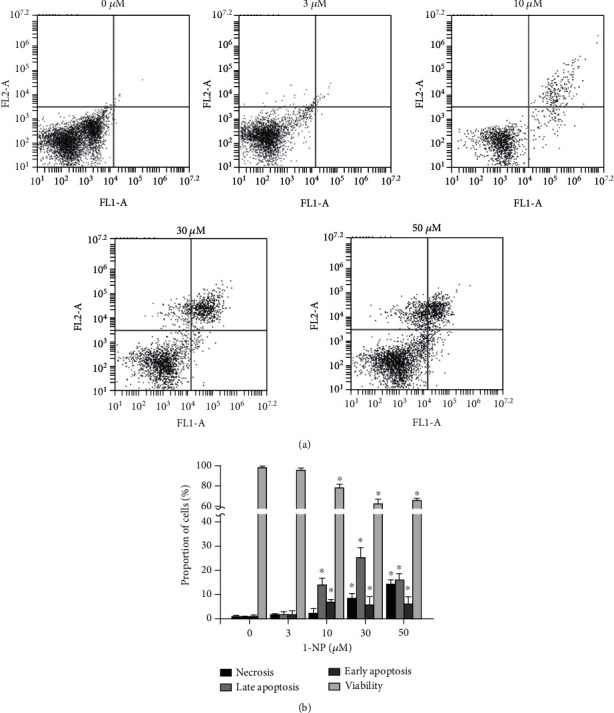
1-NP induced apoptosis and necrosis in RAW264.7 macrophages. The portion of apoptosis and necrosis was measured by Annexin V-FITC and PI assays using flow cytometry. (a) Cells were incubated with 1-NP at concentrations of 0, 3, 10, 30, and 50 *μ*M for 24 h at 37°C. The upper left quadrant (Annexin V−/+) is representative of necrosis; the upper right and lower right quadrants (Annexin V+/PI+ and Annexin V+/PI−) are representatives of apoptosis; and the lower left quadrant (Annexin V−/PI−) is representative of living cells. (b) Quantitatively, the percentage of necrotic cells, viable cells, and apoptotic cells was calculated and analyzed. Data are expressed as mean ± SD (*n* = 5). ^∗^*P* < 0.05 was considered significant compared with the control group.

The effects of 1-NP-induced apoptosis and necrosis in RAW264.7 cells were assessed using FITC-Annexin V and PI double staining. Annexin V detects phosphatidylserine externalization during apoptosis, and PI stains detect necrotic and dead cells. 1-NP induced apoptosis and necrosis in a concentration-dependent manner (Figures 2(a) and 2(b)). After 24 h, a concentration of 10 *μ*M of 1-NP significantly increased early and late apoptosis (*P* < 0.05). A concentration of 30 *μ*M of 1-NP significantly increased necrosis (*P* < 0.05).

### 3.3. Effects of 1-NP on Mitochondrial Dysfunction in RAW264.7 Macrophages

**Figure 3:**
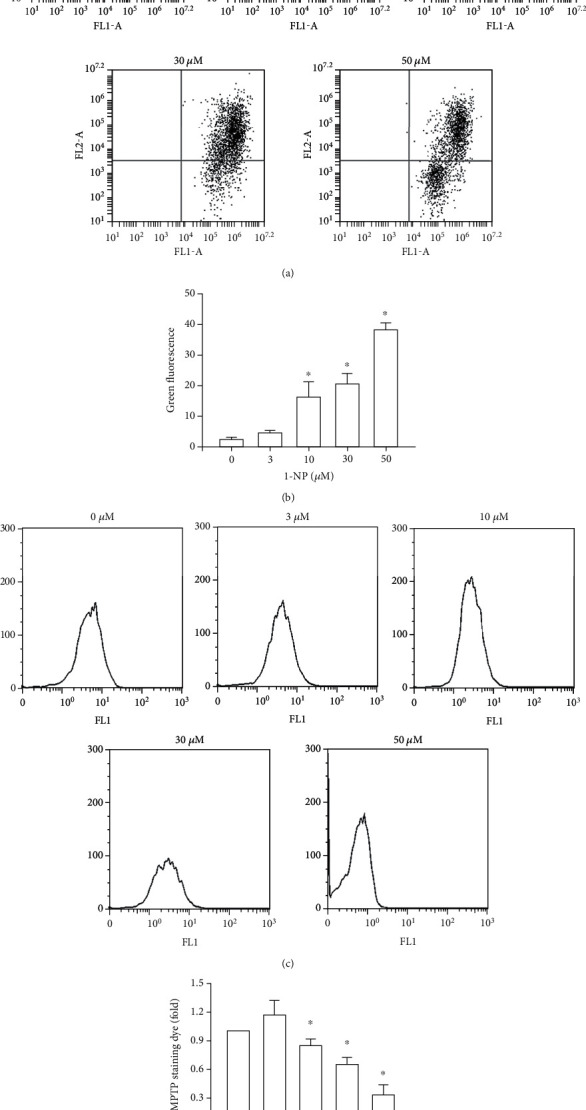
1-NP induced mitochondria dysfunction in RAW264.7 macrophages. The portion of mitochondria dysfunction was measured by JC-1 assays using flow cytometry. (a) After cell treatment, the portion of MMP was measured using the JC-1 assay. (b) Quantitatively, the percentage of MMP downregulation cells was calculated and analyzed. The MPTP opening was measured by flow cytometry. (c) After cell treatment, the histogram of MPTP opening was measured using the MPTP assay kit. (d) The change in fold of MPTP opening cells between the treated and control groups was calculated. Data are expressed as mean ± SD (*n* = 5). ^∗^*P* < 0.05 was considered significant compared with the control group.

Mitochondrial dysfunction is assessed by MMP and MPTP assays. The effects of 1-NP on mitochondrial dysfunction in RAW264.7 macrophages were investigated using JC-1, a mitochondrion-selective aggregate dye. Active mitochondria with a high membrane potential exhibited red fluorescence, and dysfunctional mitochondria with a low membrane potential exhibited green fluorescence. 1-NP induced downregulration of MMP in a concentration-dependent manner After 24 h, a concentration of 10 *μ*M of 1-NP significantly decreased the MMP (*P* < 0.05, Figure 3). 1-NP induced upregulation of the MPTP opening in a concentration-dependent manner. After 24 h, a concentration of 10 *μ*M of 1-NP significantly increased the MPTP opening (*P* < 0.05, Figure 3). These results indicated that 1-NP induced mitochondrial dysfunction in a concentration-dependent manner. After 24 h, a concentration of 10 *μ*M of 1-NP significantly increased the mitochondrial dysfunction.

### 3.4. Effects of 1-NP on Subcellular Fraction Translocation of AIF in RAW264.7 Macrophages

**Figure 4:**
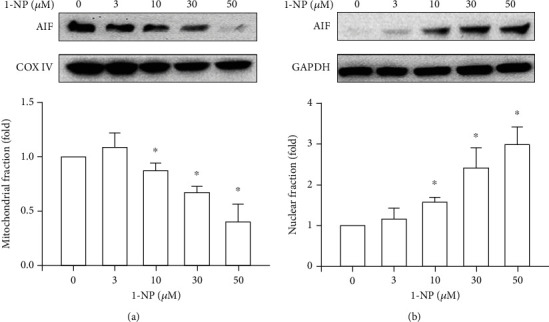
1-NP-induced nuclear translocation of AIF in RAW264.7 cells. The expression of AIF in mitochondria (a) and nucleus (b) was measured by western blot assay after treated with 1-NP for 24 h. The change in fold of AIF expression between the treated and control groups was calculated. Results are expressed as means ± SD (*n* = 3). ^∗^*P* < 0.05 was considered significant as compared with the control group.

The translocation of AIF from the mitochondria to the nucleus is critical in the caspase-independent mitochondrial apoptosis pathway. The levels of AIF in the mitochondria and nucleus were measured using western blot assay in RAW264.7 cells incubated with 1-NP. Levels of AIF in the mitochondria were reduced by 1-NP in a concentration-dependent manner and significantly increased at 10 *μ*M (*P* < 0.05; Figure 4). The effects of 1-NP on AIF levels in the nucleus were concentration dependent, with AIF significantly increased at 10 *μ*M (*P* < 0.05).

### 3.5. Effects of 1-NP on Expression Level of Bcl-2 Family Proteins in RAW264.7 Macrophages

**Figure 5:**
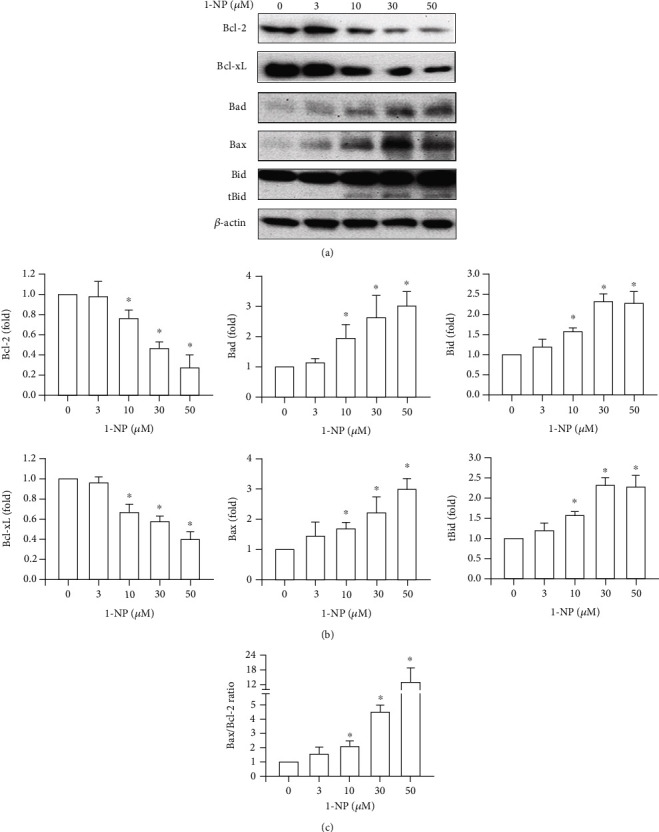
1-NP-induced change in the expression of Bcl-2 family proteins in RAW264.7 cells. (a) The expression of Bcl-2 family proteins was measured by western blot assay after being treated with 1-NP for 24 h. (b) The change in fold of Bcl-2 family expression between the treated and control groups was calculated. (c) The change in the value of Bax/Bcl-2 ratio. Results are expressed as means ± SD (*n* = 3). ^∗^*P* < 0.05 was considered significant as compared with the control group.

This study examined the regulation of mitochondrial integrity by Bcl-2 family proteins, with particular attention to the controlled release of AIF and ROS involved in caspase-independent cell death [23, 24]. The effects of 1-NP on the expression of Bcl-2 family proteins in RAW264.7 cells are illustrated in Figure 5. The levels of Bcl-2 and Bcl-xL were reduced by 1-NP in a concentration-dependent manner, and a significant effect was indicated at concentrations ≥ 10 *μ*M (*P* < 0.05). By contrast, the levels of Bad, Bax, Bid, and tBid were increased by 1-NP in a concentration-dependent manner and a significant effect was indicated at concentrations ≥ 10 *μ*M (*P* < 0.05). Moreover, the value of Bax/Bcl-2 ratio was increased by 1-NP in a concentration-dependent manner and significant effect indicated at concentration ≥ 10 *μ*M (*P* < 0.05).

### 3.6. Effects of 1-NP on Intracellular ROS Generation in RAW264.7 Macrophages

**Figure 6:**
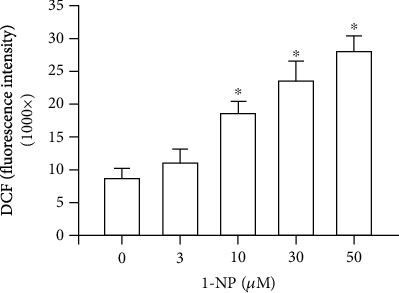
1-NP induced ROS generation in RAW264.7 macrophages. Data are expressed as mean ± SD (*n* = 5). ^∗^*P* < 0.05 is considered significant compared with the control group.

Intracellular ROS generation results in apoptosis through mitochondrial dysfunction [14]. After RAW264.7 cells were treated with 1-NP for 24 h, the intracellular ROS generation increased in a concentration-dependent manner and a significant effect was indicated at concentrations ≥ 10 *μ*M (*P* < 0.05, Figure 6).

### 3.7. Effects of 1-NP on AOE Activities in RAW264.7 Macrophages

**Figure 7:**
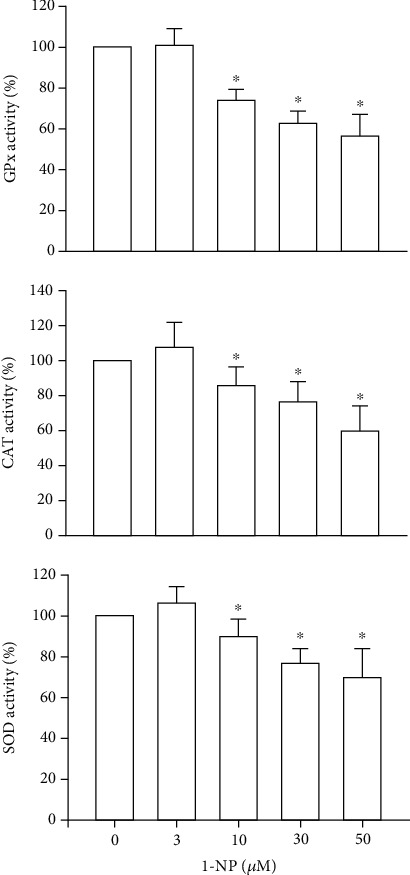
1-NP inhibited the activities of AOEs, including GPx, SOD, and GPx, in RAW264.7 macrophages. The AOE activities were measured by colorimetric assay kit after cells were treated with 1-NP. Results are expressed as means ± SD (*n* = 4). ^∗^*P* < 0.05 was considered significant compared with controls.

The activation of AOEs including GPx, SOD, and CAT plays a critical role in the control of intracellular ROS generation [14]. The activation of AOEs in RAW264.7 cells treated with 1-NP at various concentrations for 24 h was monitored using AOE assay kits. GPx, SOD, and CAT activities were induced by 1-NP in a concentration-dependent manner, and a significant effect was noted at concentrations ≥ 10 *μ*M (*P* < 0.05; Figure 7).

### 3.8. Effects of 1-NP on the AMPK/Nrf-2/HO-1 Pathway in RAW264.7 Macrophages

**Figure 8:**
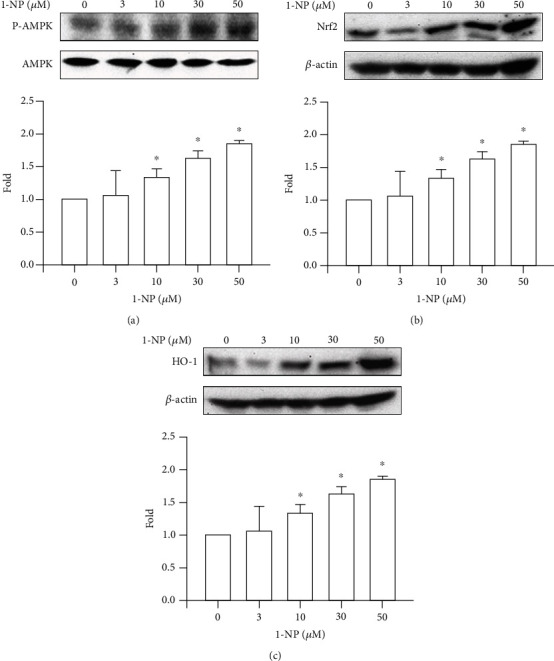
1-NP induced the activation of AMPK/Nrf-2/HO-1 pathway in RAW264.7 macrophages. AMPK phosphorylation (a), Nrf-2 expression (b), and HO-1 expression (c) were measured by colormetric assay kit after cells were treated with 1-NP. The change in fold of AMPK phosphorylation, Nrf-2 expression, and HO-1 expression between the treated and control groups was calculated. Results are expressed as means ± SD (*n* = 3). ^∗^*P* < 0.05 was considered significant as compared with the control group.

HO-1 is an antioxidative protein involved in the resolution of inflammation. Its expression is regulated by the nuclear translocation of Nrf-2. The nuclear accumulation of Nrf-2 is induced by AMPK phosphorylation. After RAW264.7 cells were treated with 1-NP for 24 h, AMPK phosphorylation, Nrf-2 expression, and HO-1 expression were induced by 1-NP in a concentration-dependent manner; a significant effect was noted at concentrations ≥ 10 *μ*M (*P* < 0.05; Figure 8).

## 4. Discussion

Air pollution can harm the environment in the forms of haze, acid rain, eutrophication, wildlife injury, and global climate change. Around the globe, diesel exhaust is a major contributor to air pollution, which can cause health problems, such as allergies, neurodegenerative diseases, and cardiovascular disease [25–27]. 1-NP and its urinary metabolites have been proposed as markers for diesel exhaust from traffic- and factory-related diesel particulate matter [28]. The mutagenic capability of 1-NP is reduced by alveolar macrophages through phagocytosis [29]. A previous study proposed that the cellular viability of RAW264.7 cells was weakly but significantly reduced by 1-NP exposure at 80 nM for 24 h [30]. In our previous study, it was found that cytotoxicity was induced by 1-NP in the concentration- and time-dependent manner. The induction was significant when the cells were treated with 3 *μ*M 1-NP for 48 h or 10 *μ*M 1-NP for 6 h [21]. The results from the present study support existing evidence that 1-NP reduces the viability of RAW264.7 cells. Furthermore, our data suggest that 1-NP reduces the viability of RAW264.7 cells in a concentration- and time-dependent manner.

Apoptosis is a major form of cell death and occurs as a defense mechanism of the immune system when cells are exposed to harmful substances [31, 32]. Previous studies have shown that 1-NP causes apoptosis in human alveolar-basal epithelial A549 cells, human bronchial epithelial BEAS-2B cells, and mouse hepatoma Hepa1c1c7 cells [12, 33, 34]. Necrosis is a type of irreversible cell injury and results in cell death [32]. Previous studies have found that 1-NP causes necrosis in Hepa1c1c7 cells and BEAS-2B cells [33, 34]. Our results also indicate that 1-NP induced apoptosis and necrosis in RAW264.7 cells. Moreover, 1-NP-induced apoptosis was observed in RAW264.7 cells at a lower concentration than 1-NP-induced necrosis. The extent of apoptosis, including early- and late-phase apoptosis, was higher than the extent of necrosis. After RAW264.7 cells were treated with 1-NP at 10 *μ*M for 24 h, cell viability decreased and apoptosis increased significantly. These results suggest that apoptosis is the major form of cell death in 1-NP-treated RAW264.7 cells.

Mitochondrial dysfunction is a critical factor in macrophage apoptosis [20, 35]. During mitochondrial dysfunction, the dissipation of mitochondrial membrane potential and loss of mitochondrial membrane integrity were observed in macrophages after exposure to apoptotic stimuli [20, 35]. AIF, a mammalian-soluble protein containing flavin adenine dinucleotide, is a nicotinamide adenine dinucleotide-dependent oxidoreductase located in the mitochondrial intermembrane space [36]. In physiological conditions, AIF plays a crucial role in mitochondrial bioenergetics. During apoptosis, loss of mitochondrial membrane integrity results in the translocation of AIF from the mitochondria to the nucleus [37]. The degradation complex formed by AIF and related proteins promotes apoptotic DNA damage [36, 37]. To the best of our knowledge, no previous studies have proposed that 1-NP decreases the mitochondrial membrane potential in macrophages. However, a previous study reported that the nuclear translocation of AIF from the cytosol to the nucleus occurred after exposure to 1-NP in Hepa1c1c7 cells, as indicated in immunocytochemical analysis [38]. The nuclear translocation of AIF pertains mainly to the elucidation of 1-NP-treated RAW264.7 cells. The present study demonstrated that the nuclear translocation of AIF was induced by 1-NP in a concentration-dependent manner in RAW264.7 cells. These results indicate that 1-NP induces apoptosis through the dissipation of mitochondrial membrane potential and the nuclear translocation of AIF due to the disruption of mitochondrial membrane.

The permeabilization of the mitochondrial membrane and the release of intermembrane space proteins (including AIF) are mediated by Bcl-2 family proteins [39, 40]. The Bcl-2 family proteins can generally be divided into three groups based on their primary function: antiapoptotic proteins, which include Bcl-2 and Bcl-xL; proapoptotic pore-formers, including Bax; and proapoptotic BH3-only proteins, which include a sensitizer protein (Bad) and activator proteins (Bid and tBid) [41]. After cells are incubated with apoptosis inducers, the activator BH3-only proteins (Bid and tBid) translocate to the mitochondrial membrane and increase their affinity for the pore former, Bax. Bax causes pore formation on the mitochondrial membrane and the leakage of AIF and other soluble proteins from the intermembrane space [39, 41]. The interaction between the activator BH3-only proteins and the pore-former protein is suppressed by antiapoptotic proteins (Bcl-2 and Bcl-xL). The sensitizer BH3-only protein, Bad, binds to and inhibits the activities of Bcl-2 and Bcl-xL [39, 41]. A previous study proposed that 1-NP induces the mRNA expression of Bax in a concentration-dependent manner in A549 cells [42]. The present study examined the expression of the Bcl2 family in 1-NP-treated RAW264.7 macrophages. We found that 1-NP induced expressions of Bid, tBid, Bax, and Bad in a concentration-dependent manner. By contrast, 1-NP reduced expressions of the antiapoptotic proteins, Bcl-2 and Bcl-xL, in a concentration-dependent manner. Crucially, the parallel trends are suitable in mitochondrial dysfunction, in AIF leakage, and in 1-NP-treated RAW264.7 macrophages. These results indicate that 1-NP induced mitochondrial dysfunction and AIF leakage by changing the expression of Bcl-2 family proteins.

Oxidative stress, triggered by mitochondrial dysfunction, has been shown to play a critical role in apoptosis [43, 44]. Overgeneration of ROS leads to high oxidative stress and encourages AOE and HO-1 [45, 46]. HO-1 degrades heme to biliverdin, which is subsequently converted to bilirubin, an antioxidant that scavenges and neutralizes ROS [46]. SOD catalyzes the reduction of superoxide anions to hydrogen peroxide. GPx and CAT catalyze the reduction of hydrogen peroxide to water and oxygen [45]. Nrf-2 is an important transcription factor that regulates the expressions of AOEs, such as HO-1 and GPx [47]. AMPK is an upstream factor for the reduction of oxidative stress in macrophages [48]. Intracellular ROS generation is induced by 1-NP in the extravillous trophoblast HTR8/SVneo cells, A549 cells, and BEAS-2B cells in *Tigriopus japonicus* [12, 49, 50]. To clarify the ROS generation and regulative mechanism induced by 1-NP in macrophages, we measured the production of intracellular ROS in RAW264.7 cells exposed to 1-NP. We found that 1-NP induced ROS generation; reduced AOE activity; and downregulated AMPK phosphorylation, Nrf-2 expression, and HO-1 expression. Based on these findings, we suggest that 1-NP induces ROS by causing mitochondrial dysfunction and reducing AOE activity. Further, we propose that 1-NP induces the activation of the AMPK/Nrf-2/HO-1 pathway to reduce oxidative damage in macrophages.

However, there are the limitations in the present study. First, RAW264.7 cells are the mouse macrophage cell line not the human macrophage. Undoubtedly, direct measurement of the toxic mechanism of 1-NP in human macrophage would be ideal, but the sampling of human macrophage raises major ethical concerns and therefore is not suitable for performance. And then, we proposed that the toxic effect of 1-NP was via apoptosis. On the other hand, toxic effect induced by 1-NP might be through other toxic pathways such as ferroptosis, necroptosis, and autophagy. Therefore, we will research on toxic pathways and relative mechanisms in our future studies. Finally, there are few studies to support that clinical disease associated with macrophage toxicity and activity induced by 1-NP. In the future work, we will research on 1-NP-induced macrophage dysfunction result in diseases, including atherosclerosis, diabetes, and inflammatory bowel disease in the differential animal models.

**Figure 9:**
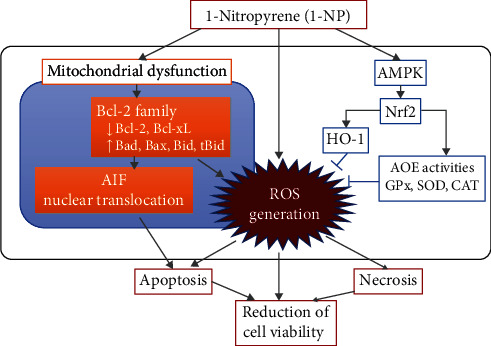
Schemes of the mechanism of the 1-NP-induced apoptosis and cytotoxicity in RAW264.7 cells. After RAW264.7 macrophages were incubated with 1-NP, it caused downregulation of cell viability via upregulation of apoptosis. 1-NP induced apoptosis by inducing AIF nuclear translocation, which was caused by mitochondrial dysfunction. Mitochondrial dysfunction induced by 1-NP occurred due to changes in the expression of BCL-2 family proteins, including downregulation of Bcl-2 and Bcl-xL and upregulation of Bad, Bax, Bid, and tBid. 1-NP induced ROS generation by mitochondrial dysfunction and reducing AOE activity. Additionally, 1-NP treatment led to the activation of the AMPK/Nrf-2/HO-1 pathway due to high levels of oxidative stress. These findings suggested that downregulation of cell viability induced by 1-NP via upregulation of apoptosis was due to mitochondrial dysfunction, AIF nuclear translocation, ROS generation, AOE activity reduction, and AMPK/Nrf-2/HO-1 pathway activation.

In conclusion, the present study found that 1-NP treatment led to downregulation of cell viability and upregulation of apoptosis in RAW264.7 macrophages (Figure 9). The findings indicate that 1-NP led to apoptosis by inducing AIF nuclear translocation, which was caused by mitochondrial dysfunction. Our data suggest that mitochondrial dysfunction occurred due to changes in the expression of BCL-2 family proteins. In addition, 1-NP induced ROS generation by reducing AOE activity. Moreover, 1-NP treatment led to the activation of the AMPK/Nrf-2/HO-1 pathway due high levels of oxidative stress. Taken together, these results suggest that 1-NP causes downregulation of cell viability and upregulation of apoptosis due to mitochondrial dysfunction, AIF nuclear translocation, ROS generation, AOE activity reduction, and AMPK/Nrf-2/HO-1 pathway activation.

## Data Availability

The data of this manuscript entitled “1-Nitropyrene Induced Reactive Oxygen Species–Mediated Apoptosis in Macrophages through AIF Nuclear Translocation and AMPK/Nrf-2/HO-1 Pathway Activation” is under license and so cannot be made freely available. Requests for access to these data should be made to Yu-Hsiang Kuan through the following E-mail address: kuanyh@csmu.edu.tw.
